# Supplementary material for: WordCommentsAnalyzer: A windows software tool for qualitative research
Source: F1000Res. 2018 Sep 4;7:536. Originally published 2018 May 3. [Version 2] doi: 10.12688/f1000research.14819.2 (PMC6137411; doi:10.12688/f1000research.14819.2)
Supplement: Supplementary file 3 [file f1000research-7-17614-s0002.tgz › b2a79496-4ada-47c4-9682-a99ce867836d.zip!/Eight Iranian Health Journals/Journal of Education and Community Health_14.docx]

© Journal of Education and Community Health. This work is licensed under CC BY-NC 4.0 (https://creativecommons.org/licenses/by-nc/4.0/).

Volume & Issue: دوره 1 - شماره 3

Title: بررسی تأثیر برنامه آموزش پیشگیری از آسیب‌های ناشی از حوادث مدرسه‌ای در دانش آموزان دوره اول دبیرستانی شهر فامنین بر اساس مدل اعتقاد بهداشتی

http://jech.umsha.ac.ir/article-1-71-fa.html

سابقه و هدف: آسیب‌های ناشی از حوادث، یکی از مشکلات مهم بهداشتی در سراسر جهان را تشکیل می‌دهد. حوادث مدرسه‌ای یکی از انواع مهم حوادث در بین کودکان و نوجوانان می‌باشد. این مطالعه باهدف تعیین تأثیر مداخله آموزشی بر اساس مدل اعتقاد بهداشتی بر عملکرد دانش آموزان در خصوص پیشگیری از آسیب‌های ناشی از حوادث مدرسه‌ای انجام‌ شده است. مواد و روش‌ها: این مطالعه مداخله‎ای از نوع تجربی بود که در بین دانش آموزان دختر و پسر دوره اول دبیرستان شهر فامنین از اسفند 1392 تا اردیبهشت 1393 انجام شد. در این مطالعه 144 دانش‌آموز به‌طور تصادفی به دو گروه کنترل و مداخله تقسیم شدند. جهت گردآوری داده‌ها از پرسشنامه‌ای مشتمل بر سؤالات دموگرافیک و سازه‌های مدل اعتقاد بهداشتی، آگاهی و عملکرد استفاده شد. این مطالعه شامل یک پیش‌آزمون و یک پس‌آزمون در دو گروه و ارائه مداخله آموزشی پنج جلسه‎ای همراه با یک کتابچه آموزشی برای گروه مداخله بوده است. داده‌ها با آزمون‌های آماری تی‌مستقل، تی‌زوجی و کای‌دو مورد تجزیه‌وتحلیل قرار گرفتند.  یافته‌ها: بعد از مداخله آموزشی بر اساس مدل اعتقاد بهداشتی، نتایج نشان داد که میانگین نمره‌ آگاهی (0.001>P)، عملکرد (0.001>P)، حساسیت درک‌شده (0.001>P)، خودکارآمدی (0.001>P) و راهنما برای عمل (0.001>P) گروه مداخله نسبت به گروه کنترل افزایش معناداری داشت. همچنین میانگین نمره موانع درک‌شده گروه مداخله در مقایسه با گروه کنترل به‌طور معنی‌داری کاهش یافت (0.001>P). نتیجه‌گیری: نتایج مطالعه حاضر نشان داد مداخله آموزشی بر اساس مدل اعتقاد بهداشتی می‌تواند در بهبود عملکرد دانش آموزان در پیشگیری از آسیب‌ها مؤثر باشد.

Title: اثربخشی مداخله آموزشی مبتنی بر مدل اعتقاد بهداشتی جهت توانمندسازی دامداران در برابر رفتارهای پرخطر تب مالت

http://jech.umsha.ac.ir/article-1-70-fa.html

سابقه و هدف: تب مالت یکی از شایع‌ترین بیماری‌های مشترک انسان و حیوان است. آموزش می‌تواند در پیشگیری از این بیماری مؤثر باشد. هدف از انجام این مطالعه ارزیابی اثربخشی مداخله آموزشی مبتنی بر مدل اعتقاد بهداشتی جهت توانمندسازی دامداران در برابر رفتارهای پرخطر تب مالت در شهرستان چاراویماق بود. مواد و روش‌ها: این پژوهش نیمه‌تجربی در سال 1393 در چاراویماق آذربایجان شرقی اجرا شد. تعداد 200 دامدار با روش نمونه‌گیری تصادفی طبقه‌ای جهت شرکت در این مطالعه انتخاب شدند. داده ها با استفاده از پرسشنامه محقق ساخته‌ای که شامل اطلاعات جمعیت شناختی، آگاهی و سازه‌های مدل اعتقاد بهداشتی بود، جمع‌آوری گردید. سپس جلسات آموزشی برای گروه مداخله طراحی و برگزار شد. 3 ماه پس از اجرای مداخله، اطلاعات از هر دو گروه‌ جمع‌آوری و با استفاده از آزمون های آماری من‌ویتنی و ویلکاکسون تجزیه‌وتحلیل گردید.  یافته‌ها: قبل از مداخله، دو گروه از نظر میانگین نمرات آگاهی، سازه‌های مدل اعتقاد بهداشتی (حساسیت، شدت، موانع، منافع درک‌شده و خودکارآمدی) و رفتارهای پیشگیری‌کننده از تب مالت، تفاوت معنی‌داری نداشتند، اما بعد از مداخله آموزشی، در گروه مداخله اختلاف معنی‌داری نسبت به گروه کنترل مشاهده گردید (0.05>P).  نتیجه‌گیری: مشارکت افراد تأثیرگذار در برنامه‌های مداخلاتی همراه با استفاده از تئوری‌های آموزشی می‌تواند اثربخشی بیشتری در اصلاح رفتارهای پرخطر در افراد داشته باشد، لذا چنین برنامه‌هایی باید در سطح وسیعی اجرا گردد.

Title: مصرف منظم صبحانه و پیش‌بینی‌کننده‌های آن بر اساس تئوری شناختی اجتماعی در میان دانشجویان دختر دانشگاه علوم پزشکی همدان

http://jech.umsha.ac.ir/article-1-51-fa.html

سابقه و هدف: علیرغم فواید شناخته‌شده مصرف منظم صبحانه، میزان حذف این وعده غذایی بالا است. مطالعه حاضر باهدف شناسایی عوامل مؤثر در مصرف صبحانه در میان دانشجویان دختر دانشگاه علوم پزشکی همدان و با استفاده از متغیرهای نظریه شناختی اجتماعی انجام گردید.  مواد و روش‌ها: در این مطالعه مقطعی، 423 دانشجوی دختر از دانشکده‌های مختلف دانشگاه علوم پزشکی همدان موردبررسی قرار گرفتند. نمونه‌گیری به‌صورت تصادفی چندمرحله‌ای بود. تواتر مصرف صبحانه و متغیرهای نظریه شناختی اجتماعی شامل آگاهی، امید و انتظار پیامد، یادگیری مشاهده‌ای، حمایت اجتماعی و خودکارآمدی با استفاده از یک پرسشنامه خود ایفا اندازه‌گیری شد. تحلیل داده‌ها با استفاده از نسخه 16 نرم‌افزار SPSS و با به‌کارگیری آزمون‌های کای‌دو، آنالیز همبستگی و رگرسیون خطی انجام شد. یافته‌ها: 24 درصد از دانشجویان گزارش کردند که به‌صورت منظم صبحانه مصرف می‌کنند. میزان حذف صبحانه در میان دانشجویان 10 درصد و میانگین مصرف 4.2 بار در هفته بود. از بین سازه‌های تئوری شناختی اجتماعی، خودکارآمدی (P<0.001) و حمایت اجتماعی (P<0.001) پیش‌بینی‌کننده معنی‌دار مصرف صبحانه در میان دانشجویان بودند. این دو متغیر درمجموع توانستند 64 درصد از تغییرات رفتار مصرف صبحانه را تبیین نماید. نتیجه‌گیری: نتایج این مطالعه نشان داد که در طراحی مداخلات تغذیه‌ای در جهت افزایش مصرف صبحانه در دانشجویان دختر باید خودکارآمدی و حمایت اجتماعی را مورد هدف قرارداد.

Title: پیشگویی‌کننده‌های مصرف سیگار در نوجوانان پسر شهر همدان بر اساس نظریه رفتار برنامه‌ریزی‌شده

http://jech.umsha.ac.ir/article-1-64-fa.html

سابقه و هدف: عوامل شناختی مرتبط با رفتارهای اجتماعی نقش تعیین‌کننده‌ای در تصمیم نوجوانان برای شروع مصرف سیگار دارند. بنابراین، مطالعه حاضر باهدف بررسی پیشگویی‌کننده‌های مصرف سیگار در بین نوجوانان شهر همدان با بهره‌گیری از نظریه رفتار برنامه‌ریزی‌شده انجام گردید.  مواد و روش‌ها: این مطالعه توصیفی تحلیلی بر روی 810 نفر از نوجوانان پسر دبیرستانی شهر همدان با استفاده از روش نمونه‌گیری چندمرحله‌ای انجام شد. ابزار گردآوری اطلاعات شامل اطلاعات جمعیت‌شناختی و پرسشنامه مبتنی بر سازه‌های نظریه رفتار برنامه‌ریزی‌شده بود که به روش خودگزارش‌دهی توسط شرکت‌کنندگان تکمیل گردید. داده‌ها با استفاده از ویرایش 18 نرم‌افزار SPSS و ویرایش 18 نرم‌افزار Amos با به‌کارگیری آزمون‌های همبستگی پیرسون و شاخص‌های برازش مدل مورد تجزیه‌وتحلیل قرار گرفت.  یافته‌ها: در کل 17.2 درصد از نوجوانان پسر به مصرف سیگار درگذشته اشاره نمودند. سازه‌های کنترل رفتاری درک‌شده، هنجارهای انتزاعی و نگرش به ترتیب بهترین پیش‌بینی‌کننده قصد مصرف سیگار بودند(0.001>P). همچنین کنترل رفتاری درک‌شده (&beta=-0.59، P<0.001) نسبت به قصد رفتاری (&beta=0.11، P<0.001) از قدرت پیش‌بینی کنندگی بالاتری برای پیش‌بینی رفتار برخوردار بود. در مدل معادلات ساختاری، 32 درصد از واریانس قصد رفتاری و 50 درصد از واریانس رفتار توسط سازه‌های نظریه تبیین گردید.  نتیجه‌گیری: نتایج حاکی از نقش کم‌رنگ قصد رفتاری در توصیف رفتار مصرف سیگار در بین نوجوانان پسر می‌باشد. بنابراین توجه به سایر عوامل روان‌شناختی مؤثر در فرآیند اخذ تصمیم برای مصرف دخانیات در بین نوجوانان ضروری به نظر می‌رسد.

Title: بررسی رابطه بین هوش هیجانی و مهارت ‌های ارتباطی دانش ‌آموزان دبیرستانی شهر همدان بر مبنای تئوری حمایت اجتماعی

http://jech.umsha.ac.ir/article-1-45-fa.html

سابقه و هدف: اهمیت توجه به هوش هیجانی در نوجوانان نسبت به گذشته افزایش چشمگیری داشته و باید مهارت‏ های گوناگون در آنان تقویت گردد؛ ازجمله این مهارت‏ ها برقراری ارتباط مؤثر و کارآمد می‏ باشد. این مطالعه با هدف تعیین رابطه بین هوش هیجانی و مهارت‏ های ارتباطی دانش‏ آموزان مقطع دبیرستان شهر همدان بر اساس تئوری حمایت اجتماعی انجام شد.  مواد و روش ‏ها: این مطالعه توصیفی، تحلیلی در سال 1393 بر روی 497 نفر از دانش ‏آموزان دبیرستانی شهر همدان انجام گرفت. آزمودنی ‏ها به روش نمونه‌گیری چندمرحله‌ای انتخاب‌شده و پرسشنامه‏ مهارت های ارتباطی، هوش هیجانی و حمایت اجتماعی را تکمیل کردند. داده‏ های جمع‌آوری‌شده با نرم‌افزار SPSS-18 و با آزمون‏ های ضریب همبستگی پیرسون، اسپیرمن، آزمون تی‌مستقل و آنالیز واریانس یک‌طرفه تجزیه‌وتحلیل گردید.   یافته ‏ها: بر پایه نتایج بین هوش هیجانی و مهارت‏ های ارتباطی دانش ‏آموزان مورد بررسی ارتباط معنی‏ داری وجود داشت (P<0.05). سطح مهارت‏ های ارتباطی دانش‏ آموزانی که در این مطالعه موردبررسی قرار گرفتند در حد متوسط بوده و پژوهش حاضر برتری کلی سطح نمرات مهارت‏ های ارتباطی، هوش هیجانی و حمایت اجتماعی(به‌جز حمایت ارزیابی) در گروه دختران نسبت به پسران را نشان داد، همچنین بین هوش هیجانی و مهارت‏ های ارتباطی با سازه های حمایت اجتماعی ارتباط معنی‏ داری وجود داشت (P<0.05).  نتیجه‏ گیری: طبق یافته‏ ها جهت افزایش هوش هیجانی و ارتقاء مهارت‏ های ارتباطی نیاز به انجام مداخلات آموزشی، تهیه و تدوین بسته‏ های آموزشی جهت دانش ‏آموزان، والدین آنها و مربیان مدارس احساس می‏ شود.

Title: عوامل شناختی پیش‌بینی‌کننده ازدواج فامیلی در میان مراجعین به مراکز مشاوره ازدواج استان کهکیلویه و بویراحمد

http://jech.umsha.ac.ir/article-1-50-fa.html

سابقه و هدف: در ازدواج‌های فامیلی احتمال تولد نوزاد معلول 2 تا 3 برابر بیشتر است که این امر می‌تواند پیامدهای منفی متعددی را به دنبال داشته باشد. هدف از انجام این پژوهش، تعیین عوامل شناختی پیش‌بینی‌کننده ازدواج فامیلی بود.  مواد و روش‌ها: پژوهش حاضر یک مطالعه توصیفی به روش مقطعی بود که در میان 516 نفر از مراجعین به مراکز مشاوره قبل از ازدواج استان کهکیلویه و بویراحمد انجام گرفت. آزمودنی‌ها به روش نمونه‌گیری در دسترس، در میان چهار مرکز مشاوره قبل از ازدواج انتخاب شدند و اطلاعات به‌ وسیله پرسشنامه و به‌ صورت خود گزارش‌دهی جمع‌ آوری گردید. داده‌های جمع‌ آوری شده با نرم‌افزار SPSS-20 و با آزمون‌های آماری همبستگی پیرسون و آنالیز رگرسیون لجستیک در سطح معناداری کمتر از 0.05 تحلیل شدند. یافته‌ها: دامنه سن شرکت‌کنندگان 15 تا 30 و میانگین آن 3.96±23.43 سال بود. ازدواج 43.4 درصد از شرکت‌کنندگان به‌صورت فامیلی بود. نرم‌های انتزاعی (1.304=OR) و عوامل فرهنگی (1.244=OR) عوامل قوی‌تری در پیش‌بینی اقدام به ازدواج فامیلی بودند.   نتیجه‌گیری: با توجه به بالا بودن آمار ازدواج فامیلی در این مطالعه، ضرورت توجه بیشتر به انجام اقدامات اطلاع‌رسانی در خصوص مشاوره‌های ژنتیکی پیش از ازدواج پیشنهاد می‌شود. همچنین به نظر می‌رسد در طراحی مداخلات آموزشی توجه به نرم‌های انتزاعی و عوامل فرهنگی مرتبط با ازدواج فامیلی می‌تواند نتایج سودمندی را در این زمینه به دنبال داشته باشد.

Title: کاربرد مدل بزنف در پیش‌بینی انجام فعالیت فیزیکی پس از زایمان در بین مادران مراجعه‌کننده به مراکز بهداشتی درمانی شهر کرمانشاه

http://jech.umsha.ac.ir/article-1-62-fa.html

سابقه و هدف: مادران پس از زایمان در معرض خطر بالای زندگی کم‌تحرک بوده و احتمال بروز چاقی و دیابت نیز در این گروه بیشتر از سایرین است. بنابراین، مطالعه حاضر با هدف بررسی عوامل پیش‌بینی‌کننده انجام فعالیت فیزیکی پس از زایمان در مادران مراجعه‌کننده به مراکز بهداشتی درمانی شهر کرمانشاه با بهره‌گیری از مدل بزنف انجام گردید. مواد و روش‌ها: این مطالعه توصیفی تحلیلی و از نوع مقطعی در سال 1393 بر روی 400 نفر از مادران مراجعه‌کننده به مراکز بهداشتی درمانی شهر کرمانشاه با استفاده از روش نمونه‌گیری چندمرحله‌ای انجام شد. ابزار گردآوری داده‌ها شامل اطلاعات دموگرافیک، پرسشنامه مبتنی بر سازه‌های مدل بزنف و فرم کوتاه پرسشنامه فعالیت فیزیکی بود. داده‌ها با استفاده از ویرایش 18 نرم‌افزار SPSS و با به‌کارگیری آزمون‌های رگرسیون لجستیک، رگرسیون خطی و همبستگی پیرسون مورد تجزیه‌وتحلیل قرار گرفت. یافته‌ها: در این مطالعه 83 درصد از مادران دارای فعالیت فیزیکی سبک یا کم بودند. طبق یافته‌ها، آگاهی، نگرش و هنجارهای انتزاعی عوامل پیش‌بینی‌ کننده قصد انجام فعالیت فیزیکی بوده و 21 درصد از واریانس قصد رفتاری را تبیین می‌نمودند. همچنین قصد رفتاری بهترین پیش‌بینی‌ کننده رفتار فعالیت فیزیکی پس از زایمان بود (β=0.140، P<0.001).  نتیجه‌گیری: نتایج این تحقیق نشان‌دهنده بی‌تحرکی در اکثر مادران در دوره پس از زایمان بود. بنابراین طراحی مداخلاتی جهت ایجاد تغییر در قصد رفتاری در مادران پس از زایمان نویدبخش اثرات سودمندی خواهد بود.

Title: بررسی رابطه حمایت اجتماعی با پیروی از رژیم‌درمانی در بیماران مبتلا به پرفشاری خون مراجعه‌کننده به بیمارستان طالقانی تهران

http://jech.umsha.ac.ir/article-1-48-fa.html

سابقه و هدف: حمایت اجتماعی از عوامل مؤثر در پیروی از رژیم‌درمانی در بیماران مبتلا به پرفشاری خون می‌باشد. اما این ارتباط در جمعیت با پرفشاری خون هنوز به‌طور کامل بررسی نشده است. هدف از این پژوهش تعیین ارتباط بین حمایت اجتماعی با پیروی از رژیم‌درمانی در بیماران مبتلا به پرفشاری خون بود.  مواد و روش‌ها: مطالعه حاضر، یک مطالعه مقطعی (توصیفی-تحلیلی) می‌باشد که در آن کلیه بیماران مبتلا به پرفشاری خون (130 بیمار) کلینیک قلب بیمارستان طالقانی تهران به روش سرشماری انتخاب و وارد مطالعه شدند. ابزار گردآوری داده‌ها شامل پرسش‌نامه جمعیت شناختی، پرسش‌نامه پیروی از رژیم‌درمانی هیل بن و پرسش‌نامه حمایت اجتماعی وایکس بود. بعد از جمع‌آوری پرسش‌نامه‌ها، داده‌ها با استفاده از نرم‌افزار SPSS نسخه 16 و به‌کارگیری آزمون‌های ضریب همبستگی اسپیرمن، تی‌مستقل و آنالیز واریانس یک‌طرفه مورد تجزیه‌وتحلیل قرار گرفت. یافته‌ها: در این مطالعه 85.4 درصد از بیماران دارای پیروی متوسط از رژیم‌ درمانی، 5.4 درصد پیروی خوب و 9.2 درصد دارای پیروی ضعیف بودند. ارتباط معناداری بین حمایت اجتماعی و میزان پیروی از رژیم‌درمانی ملاحظه شد (P<0.05). بدین ترتیب که بیماران با حمایت اجتماعی متوسط و خوب، از پیروی بهتری از رژیم‌درمانی خود، نسبت به بیماران با حمایت اجتماعی ضعیف برخوردار بودند. همچنین در این مطالعه ارتباط معناداری بین سطح سواد و وضعیت تأهل با حمایت اجتماعی ملاحظه گردید (P<0.05). نتیجه‌گیری: نتایج این تحقیق نشان داد که حمایت اجتماعی باید به‌عنوان یک مؤلفه اثرگذار بر میزان پیروی از رژیم‌درمانی و سلامت بیماران با پرفشاری خون موردتوجه قرار گیرد. در نظر گرفتن حمایت اجتماعی می‌تواند منجر به بهبود وضعیت پیروی از رژیم‌درمانی در بیماران مبتلا به پرفشاری خون گردد.
